# Global Variations in Water Vapor and Saturation State Throughout the Mars Year 34 Dusty Season

**DOI:** 10.1029/2022JE007203

**Published:** 2022-10-21

**Authors:** J. A. Holmes, S. R. Lewis, M. R. Patel, J. Alday, S. Aoki, G. Liuzzi, G. L. Villanueva, M. M. J. Crismani, A. A. Fedorova, K. S. Olsen, D. M. Kass, A. C. Vandaele, O. Korablev

**Affiliations:** ^1^ School of Physical Sciences The Open University Milton Keynes UK; ^2^ Space Science and Technology Department Science and Technology Facilities Council Rutherford Appleton Laboratory Didcot UK; ^3^ Department of Physics University of Oxford Oxford UK; ^4^ Institute of Space and Astronautical Science Japan Aerospace Exploration Agency Kanagawa Japan; ^5^ Royal Belgian Institute for Space Aeronomy Brussels Belgium; ^6^ NASA Goddard Space Flight Center Greenbelt MD USA; ^7^ Department of Physics American University Washington DC USA; ^8^ Department of Physics California State University San Bernardino San Bernardino CA USA; ^9^ Space Research Institute of the Russian Academy of Sciences (IKI RAS) Moscow Russia; ^10^ Jet Propulsion Laboratory California Institute of Technology Pasadena CA USA

## Abstract

To understand the evolving martian water cycle, a global perspective of the combined vertical and horizontal distribution of water is needed in relation to supersaturation and water loss and how it varies spatially and temporally. The global vertical water vapor distribution is investigated through an analysis that unifies water, temperature and dust retrievals from several instruments on multiple spacecraft throughout Mars Year (MY) 34 with a global circulation model. During the dusty season of MY 34, northern polar latitudes are largely absent of water vapor below 20 km with variations above this altitude due to transport from mid‐latitudes during a global dust storm, the downwelling branch of circulation during perihelion season and the intense MY 34 southern summer regional dust storm. Evidence is found of supersaturated water vapor breaking into the northern winter polar vortex. Supersaturation above around 60 km is found for most of the time period, with lower altitudes showing more diurnal variation in the saturation state of the atmosphere. Discrete layers of supersaturated water are found across all latitudes. The global dust storm and southern summer regional dust storm forced water vapor at all latitudes in a supersaturated state to 60–90 km where it is more likely to escape from the atmosphere. The reanalysis data set provides a constrained global perspective of the water cycle in which to investigate the horizontal and vertical transport of water throughout the atmosphere, of critical importance to understand how water is exchanged between different reservoirs and escapes the atmosphere.

## Key Points

- Global dust storm and southern summer regional dust storm events pushed water vapor above 60 km across all latitudes
- The saturation state of the global atmosphere is investigated in a reanalysis for the first time
- Supersaturated water vapor penetrates the northern winter polar vortex

## 1 Introduction

The history of water abundance on Mars and how it has changed with time remains a major mystery. This is largely as a result of the currently incomplete understanding of how much water has escaped from the atmosphere over time and the processes through which this phenomenon occurs (Jakosky et al., 2018; Villanueva et al., 2015). More complete knowledge of how the water inventory has been altered over time on Mars requires a better understanding of the past and present martian atmospheric water cycle and the vertical mobility of water vapor. Over the past 50 years orbital and, more recently, surface observations gathered by several missions have helped to build up a more complete description of the water cycle. Mars missions have uncovered the seasonal trends in the water vapor column and investigated interannual variations, with systematic mapping of the water vapor column undertaken by TES (Thermal Emission Spectrometer; Smith, 2002, 2004), PFS (Planetary Fourier Spectrometer; Fouchet et al., 2007; Sindoni et al., 2011; Tschimmel et al., 2008), OMEGA (Observatoire pour la Minéralogie, l’Eau, les Glaces et l’Activité; Maltagliati, Titov, et al., 2011; Melchiorri et al., 2007), SPICAM (Spectroscopy for the Investigation of the Characteristics of the Atmosphere of Mars; Fedorova et al., 2006; Trokhimovskiy et al., 2015) and CRISM (Compact Reconnaissance Imaging Spectrometer for Mars; Smith et al., 2009) on several different spacecraft. One of the main outstanding questions regarding the water cycle is a complete understanding of the seasonal evolution of the vertical distribution of water vapor that has a large impact on the transport of water in the atmosphere and surface‐regolith interactions (Steele et al., 2017). The seasonal evolution of the water vapor profile has been observed until now primarily from SPICAM profiles (Maltagliati et al., 2013; Maltagliati, Montmessin, et al., 2011), which also discovered the presence of supersaturation in the atmosphere of Mars. This discovery means we know that water vapor can propagate through the hygropause more efficiently than previously thought, impacting the escape of water from the martian atmosphere. Systematic mapping of the presence of supersaturation, in particular across a complete diurnal cycle, has not yet been conducted but is important to understand how prevalent supersaturation is on Mars. Increasingly expansive water vapor profile data sets have recently been retrieved from instruments on the ExoMars Trace Gas Orbiter (TGO) spacecraft, namely the Nadir and Occultation for MArs Discovery (NOMAD; Aoki et al., 2019) and Atmospheric Chemistry Suite (ACS; Fedorova et al., 2020). Results have primarily focused on the impact of a global dust storm event on the water vapor vertical structure, along with more evidence of supersaturation. Using the latest TGO water vapor data sets that have improved resolution and spatio‐temporal mapping when compared to previous instruments, the processes affecting the water cycle and wider questions such as upper atmosphere water loss and amount of surface water ice present on Mars can now be constrained and further identified.

Observations of the martian water cycle can be spatio‐temporally sparse as a result of orbital constraints and limitations based on the technique of observation and necessitate the utilization of a global circulation model (GCM) for interpretation and understanding of the observed seasonal and latitudinal variations in a global sense. Computer models are also necessary to attempt understanding of the past climate of Mars and how it has evolved to its current state, evidence of which can be identified by orbital and rover exploration (e.g., Ehlmann & Edwards, 2014, and references therein). Microphysical models of the water cycle that can simulate nucleation on dust particles and supersaturation are now common in GCMs, with the first microphysical scheme by Navarro et al. (2014) indicating an improved match to retrievals from the TES instrument (Smith, 2002). Shaposhnikov et al. (2019) revealed a seasonal water “pump” mechanism present during the perihelion season and demonstrated how atmospheric dust controls the circulation strength and hence the abundance of water vapor at high altitudes during dust storm events. High‐altitude water observed by NOMAD during the Mars year (MY) 34 global dust storm event was subsequently investigated by Neary et al. (2020), reinforcing the view that the dust vertical distribution is a key factor in the transport of water vapor to the upper atmosphere. Understanding of how lower atmospheric variations in water vapor lead to the observed upper atmosphere escape of hydrogen (Alday et al., 2021; Chaffin et al., 2021) is key to providing complete understanding of the water cycle from surface to escape in the upper atmosphere. This has recently been investigated by Holmes et al. (2021), identifying interannual variation in the escape during regional dust storms consistent with observations from the NASA Mars Atmosphere and Volatile Evolution (MAVEN) spacecraft.

While GCMs provide a global perspective of the evolving surface and atmosphere, they do also contain errors of representation associated with computational constraints. This means it is impossible to completely represent all the physical processes occurring across the globe on local scales (10 s of kilometers and below) and therefore GCMs can never fully replicate the evolving atmosphere of Mars. Observations, on the other hand, are an important source of real information, but are restricted in space and time (e.g., TGO coverage is predominantly at high latitudes), meaning that they only provide a “snapshot” of the atmosphere and introduce an implicit bias into our understanding of atmospheric cycles through lack of spatio‐temporal coverage. Synoptic observations of the vertical distribution of water vapor in the Mars atmosphere would be preferred, but this is not currently possible.

Obtaining the best possible analysis of the global evolution of the water cycle requires combining observational data with a Mars GCM. This technique, called data assimilation, is widely used in conjunction with Earth GCMs and three different Mars reanalysis data sets are now publicly available; the Open access to Mars Assimilated Remote Soundings (OpenMARS) data set (Holmes et al., 2020), Mars Analysis Correction Data Assimilation (Montabone et al., 2014) and Ensemble Mars Atmosphere Reanalysis System (Greybush et al., 2019). The Laboratoire de Météorologie Dynamique (LMD) Mars GCM is also capable of data assimilation of temperature, dust and water ice (Navarro et al., 2014, 2017). The OpenMARS data set was created using the Open University modeling group Mars GCM, which is also capable of assimilating chemical species. The additional capability of chemical assimilation provides a more robust investigation than pure model studies that are simply compared to the observed chemical species. Chemical assimilation ensures parameters that show multiple dependencies and complex linkages (e.g., water vapor, ozone, carbon monoxide, dust opacity, and temperature) are all simultaneously constrained realistically and physically consistent when any of the parameters are altered by the assimilation method. An assimilation of TES water vapor column data (Smith, 2002, 2004) and temperature profiles (Conrath et al., 2000) has previously been used in a data assimilation study by Steele, Lewis, Patel, Montmessin, et al. (2014) that primarily investigated the important role of wave activity on the water vapor distribution. No water vapor profiles were assimilated in that study however or any of the reanalysis data sets. The latest water vapor vertical profiles data sets (Alday et al., 2021; Aoki et al., 2019; Fedorova et al., 2020; Villanueva et al., 2021) and water vapor column data (Crismani et al., 2021) retrieved by instruments on the ExoMars TGO spacecraft, when combined with a Mars GCM and multiple other existing observational data sets, creates the ideal opportunity to further our understanding of the spatiotemporal distribution of water in the atmosphere of Mars. A large knowledge gap currently exists in the coupled geographical‐vertical distribution of water, since observations lack the required spatiotemporal coverage, and the largely unconstrained vertical distribution of water vapor in pure modeling studies has not been possible to validate before now. In the special case of the dusty season of MY 34, where the vertical dust distribution is poorly represented by current models (Neary et al., 2020), assimilation has the advantage of direct inclusion of temperature and water vapor profiles that are otherwise largely impacted by the poor representation of the vertical dust distribution.

In this study we investigate the global distribution of water vapor and the saturation state of the atmosphere throughout *L*_S_ = 159–358° in MY 34 utilizing a data assimilation global reanalysis that combines together spacecraft observations from nine data sets. Sections 2 and 3 give an overview of the observations and model combined to create the water reanalysis respectively, and Section 4 provides an independent validation of the reanalysis by comparing to water vapor profiles from SPICAM independent from this study. Section 5 discusses the global transport of water vapor and saturation state of the atmosphere.

## 2 Spacecraft Observations

**Figure 1:**
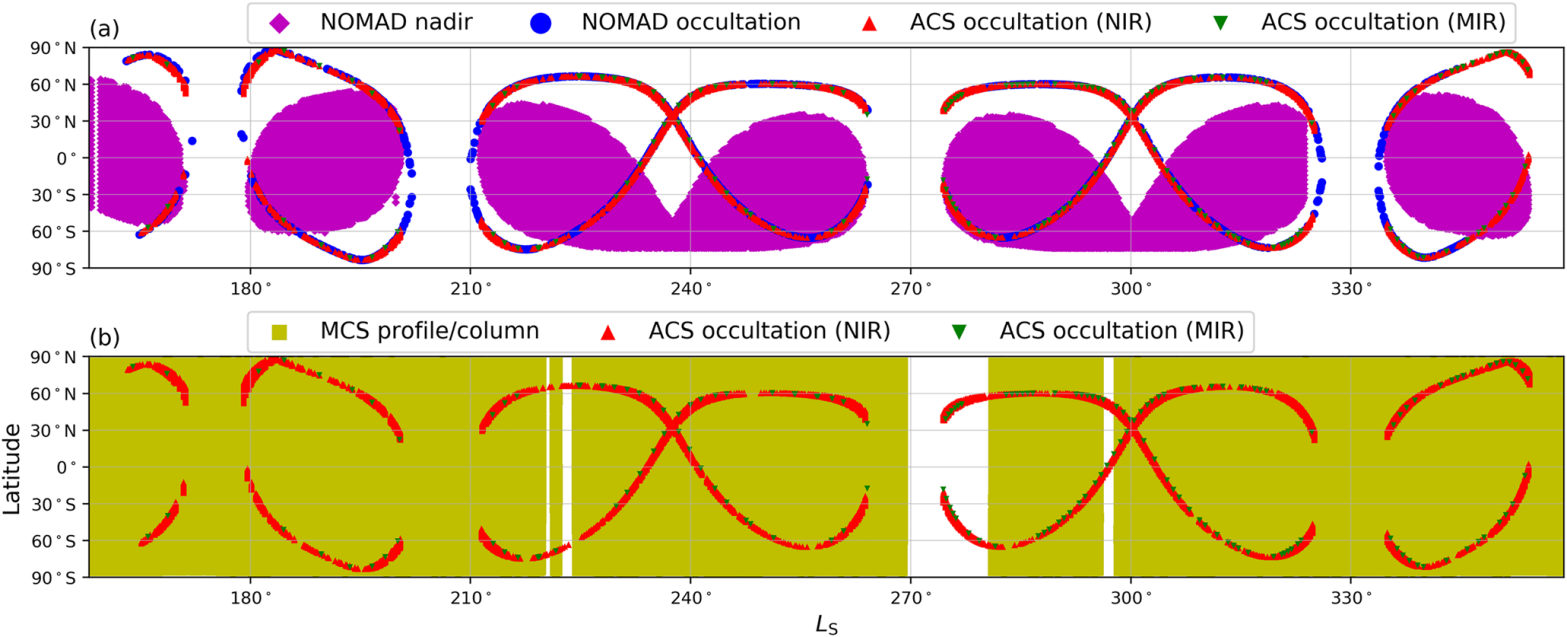
Overview of the coverage of the (a) water vapor and (b) thermal observations (Mars climate sounder provides temperature profiles and dust column) assimilated to create the global water reanalysis covering *L*_S_ = 159–358° Mars Year 34.

This study incorporates a multi‐spacecraft assimilation approach that combines a Mars GCM with retrievals from the ExoMars TGO and Mars Reconnaissance Orbiter (MRO) spacecraft currently in orbit around Mars. Differences in the orbits of these spacecraft result in a largely complementary expanded data set when combined due to the minimal overlap in data at the same local time and spatial location, with the coverage of all data sets shown in Figure 1. Data assimilation therefore is a highly efficient method in these circumstances to provide the best possible reproduction of the global atmospheric evolution during this time period.

### 2.1 ExoMars Trace Gas Orbiter

The ExoMars TGO spacecraft, a joint collaboration between the European and Russian space agencies, entered into orbit around Mars on 19 October 2016 and began science operations on 21 April 2018 after a long period of aerobraking into a near‐polar 2‐hr orbit. This particular orbit provides up to 24 occultations of the atmosphere per sol. The reanalysis created in this study includes retrievals from two of the four instruments on the TGO spacecraft; namely the NOMAD (Vandaele et al., 2018) and ACS spectrometer suites (Korablev et al., 2018).

Retrievals from all three spectrometers of the NOMAD instrument covering a wide range of spectral channels are included in the MY 34 water reanalysis. The SO (solar occultation) spectrometer (in the spectral range from 2.3 to 4.3 μm) data are used to retrieve the water vapor vertical profile up to around 100 km with (typical) vertical sampling of 1 km spanning a wide range of local times and spatial locations, with full details in Aoki et al. (2019) and Villanueva et al. (2021). Retrievals of the water vapor column (Crismani et al., 2021) and carbon monoxide column (Smith et al., 2021) from the LNO (Limb Nadir and solar Occultation) spectrometer, covering the spectral range from 2.3 to 3.8 μm, are also included in the water reanalysis. As can be seen in Figure 1, the column retrievals have greater spatial coverage throughout time than the vertical profiles that from TGO are only available at the terminator. Finally, ozone vertical profiles retrieved by UVIS (Ultraviolet and Visible Spectrometer) are incorporated into the reanalysis (Patel et al., 2021) for completeness.

Two of the three spectrometers that form the ACS instrument provide continuous spectral coverage from 0.7 to 17 μm (Korablev et al., 2018). Solar occultations from the ACS NIR (near‐infrared; 0.7–1.7 μm) spectrometer provide the water vapor and temperature vertical profile up to around 100 km with a resolution of 1–3 km, with full details in Fedorova et al. (2020). Both the water vapor and temperature ACS NIR data sets are incorporated into the reanalysis. The water vapor and temperature vertical profiles have also been retrieved by the ACS MIR (mid‐infrared) spectrometer (Alday et al., 2021; Olsen et al., 2021), with the ACS NIR and ACS MIR providing coincident profiles with good agreement (see supplementary material of Fedorova et al. (2020)). The differing boresights and sharing of occultations between the NOMAD and ACS instruments mean that the water vapor profile data sets generally alternate between different instruments, with data assimilation therefore providing an optimal way of unifying all three separate observational data sets to create a global perspective of the vertical distribution of water that is not possible by any one instrument alone.

### 2.2 Mars Reconnaissance Orbiter

The NASA MRO spacecraft began its primary science phase of monitoring the atmosphere of Mars on 24 September 2006 and remains operational. The Sun‐synchronous orbit of MRO results in observations from low to mid‐altitudes at local times around 3 a.m. and 3 p.m. though the actual local time of a given observation varies with both latitude and season.

The Mars Climate Sounder (MCS) is an infrared thermal emission radiometer containing one visible/near‐infrared channel and eight far‐infrared channels (McCleese et al., 2007). Measurements from the MCS instrument provide the temperature vertical profile up to around 85 km with a 5 km vertical resolution, with corrections in lateral temperature gradients through two‐dimensional radiative transfer methods providing improved retrievals close to the poles (Kleinböhl et al., 2017). The reanalysis produced in this study also includes a derived MCS dust column product based on extrapolation of retrieved dust profiles (Kleinböhl et al., 2017). Version 5.2 of the MCS temperature profiles and derived dust column products are included in this study, except during the global dust storm time period in which a re‐processed v5.3.2 version that incorporates additional information from the far infrared channel centered at 316 cm^−1^ is included (Kleinböhl et al., 2020).

## 3 Mars Global Circulation Model

The reanalysis created through this study incorporates a Mars GCM used by the Open University (OU) modeling group that has been developed in a collaboration between the LMD, the OU, the University of Oxford and the Instituto de Astrofísica de Andalucía. Physical parameterizations (Forget et al., 1999) and the LMD photochemical module (Lefèvre et al., 2004, 2008) both shared with the LMD Mars GCM are coupled to a spectral dynamical core and semi‐Lagrangian advection scheme (Newman et al., 2002). The advection scheme uses wind fields updated by the dynamical core every 1.2 min to determine the transport of each chemical species across the spatial domain every 12 min.

Specific physical parameterizations linked to the water cycle include the cloud microphysics package (Navarro et al., 2014) that takes into account nucleation on dust particles and supersaturation, an implementation for radiatively active clouds (Madeleine et al., 2012), a semi‐interactive two‐moment scheme to transport dust (Madeleine et al., 2011) and a thermal plume model for better representation of turbulence in the planetary boundary layer (Colaïtis et al., 2013).

The reanalysis produced by this study utilizes the Mars GCM truncated at wavenumber 31, resulting in a 5° longitude‐latitude grid in the horizontal for physical variables, with 70 vertical sigma levels that can extend to around 100 km.

### 3.1 Data Assimilation Scheme

To combine all of the retrieval data sets with the Mars GCM, the Analysis Correction (AC) scheme (Lorenc et al., 1991) is used with necessary parameters adapted to martian conditions. This particular data assimilation scheme has previously been used to investigate several martian atmosphere scientific topics (Lewis & Barker, 2005; Montabone et al., 2005, 2006, 2014; Montabone et al., 2015; Steele, Lewis, Patel, Montmessin, et al., 2014). SPICAM data have previously been assimilated to investigate features of the ozone cycle (Holmes et al., 2018) and MCS data to study the radiative effect of water ice clouds (Steele, Lewis, & Patel, 2014) and surface warming during the MY 34 global dust storm (Streeter et al., 2020). More recent studies have also moved to multi‐instrument assimilation studies, with MCS and CRISM data combined with the Mars GCM to explore the carbon monoxide cycle (Holmes et al., 2019) and also MCS and ACS data combined to investigate the asymmetry of the polar vortices (Streeter et al., 2021) and super‐rotation of the atmosphere (Rajendran et al., 2021). The same data assimilation scheme has also produced the OpenMARS data set (Holmes et al., 2020), a publicly available global record of martian weather from 1999 to 2015.

The AC scheme is a form of successive corrections in which analysis steps are interleaved with each model dynamical time step. The modified successive corrections equation applied for any retrieval assimilated by the scheme is identical to that described in Holmes et al. (2018). The analysis increments are spread from the observation locations to the surrounding model grid points in each analysis step after splitting the equation into separate vertical and horizontal stages (the background error covariance matrix is also factored into a vertical covariance and horizontal correlation). Derivation of multi‐variate increment fields for dynamical balance follows, where applicable (e.g., after assimilating temperatures, balanced thermal wind increments are applied).

For chemical species, some of which can display rapid variations on much shorter timescales, the time window for insertion of observations is adjusted to take this into account. A time window of 6 hr was found to be optimal for water vapor column data and 7 hr (6 hr before an observation's valid time until 1 hr after) for carbon monoxide column as a result of the longer lifetime of carbon monoxide in the atmosphere. For the assimilation of water vapor (and ozone) profiles, a 3 hr time window is used as a result of sharp variations that can occur in water vapor and ozone around the terminator insertion time period. Background vertical correlations are approximated using the same Gaussian function and parameter values chosen for water ice in Steele, Lewis, and Patel (2014), which allows spreading of data to at most two model levels outside the bounds of the vertical profile coverage, since water vapor can rapidly vary based on local saturation conditions.

When assimilating water vapor profiles, only the water vapor is directly adjusted but the water ice may also be indirectly altered in the next physics timestep based on the assimilated water vapor abundance. For example, if the assimilation boosts the local water vapor abundance this could potentially result in an increased water ice abundance at a later timestep if the temperature drops below the condensation temperature. Impacts of water vapor assimilation on the water ice distribution will be the subject of a future paper. The dust profile is also potentially indirectly altered in the assimilation step, for example, an increase in water ice in a later timestep from a boosted water vapor abundance (via data assimilation) would result in an increased number of cloud condensation nuclei removed from the atmosphere and in turn a decrease in local dust. The reanalysis does not conserve water during the assimilation step, so extrapolations of the long term inventory of water from the reanalysis decrease in confidence as you extrapolate further in time from that covered by the reanalysis.

## 4 Validation Against Independent SPICAM Retrievals

To validate the water vapor distribution produced by the reanalysis, the water vapor reanalysis was compared against SPICAM data that was not assimilated. An added advantage of data assimilation is that the reanalysis can be compared to retrievals that are not necessarily in the same location as the retrievals combined with the Mars GCM, but nevertheless are still influenced by assimilation of temperature profiles, dust column, and potentially even water vapor profiles in the past. The SPICAM instrument on the Mars Express spacecraft is still operational and has retrieved water vapor profiles in multiple MYs that are fully detailed in Fedorova et al. (2021), along with the data availability of the water profiles used for this validation. Only 145 water vapor profiles are available for comparison during the time period that the reanalysis is produced over (*L*_S_ = 159–358° MY 34), compared to 1,173 water vapor retrievals from NOMAD (Aoki et al., 2019). The lower number of SPICAM water vapor profiles makes it less suitable for assimilation, but provides an extremely useful data set against which to validate the reanalysis.

**Figure 2:**
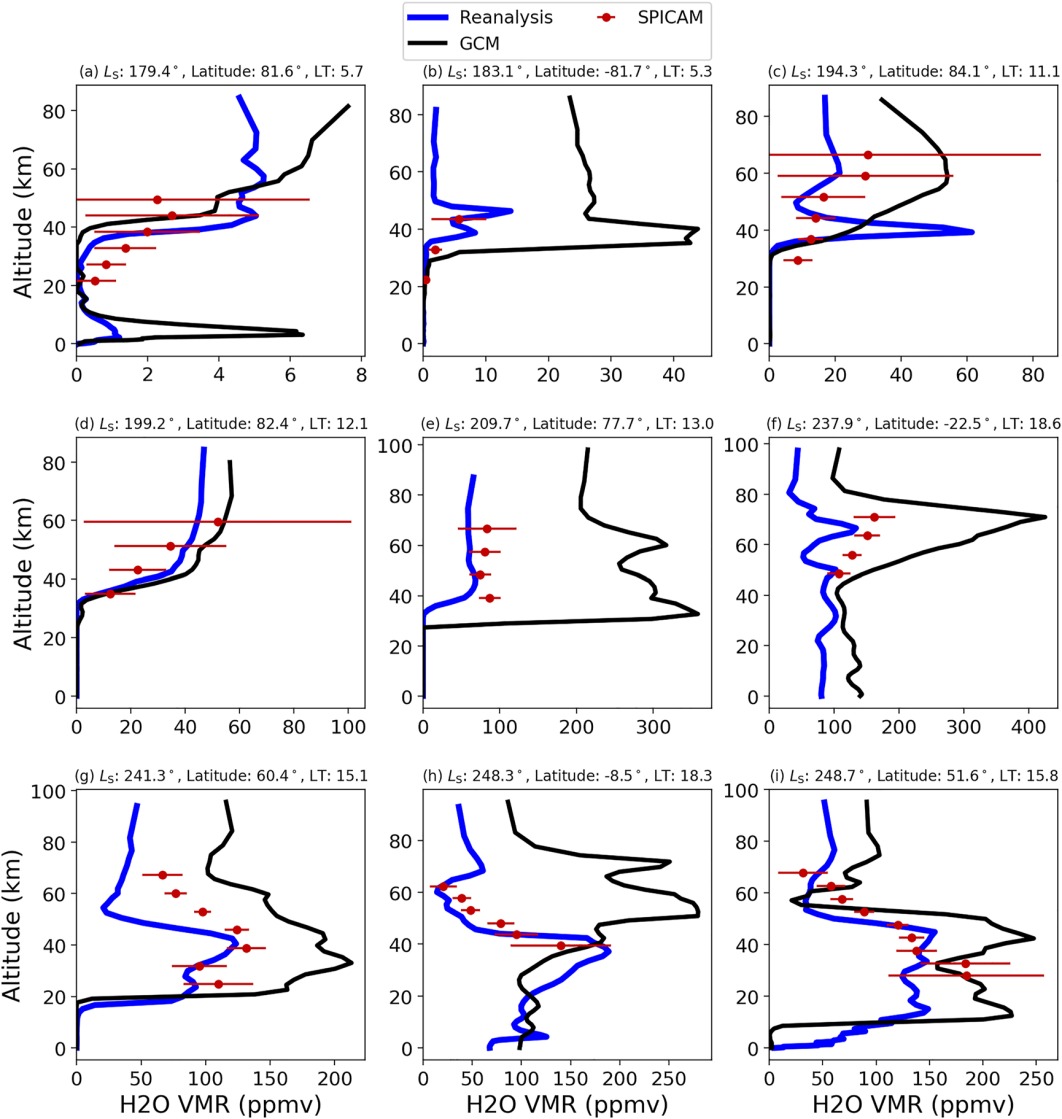
Comparison of water vapor profiles from the Spectroscopy for the investigation of the characteristics of the atmosphere of Mars instrument (red) with independent coincident water vapor profiles in the reanalysis (blue) and a free‐running global circulation model (black). Red horizontal bars indicate the uncertainty on each altitude point of the retrieval.

Figure 2 displays a comparison of SPICAM profiles to the reanalysis and a free‐running GCM at the closest spatial location and local time. SPICAM retrievals in this data set cover *L*_S_ = 166–248° for MY 34 and are located predominantly at polar regions earlier in the time period before moving towards more equatorial locations. In the comparison with the earlier SPICAM retrievals, the general trend at polar latitudes is for a decrease in water vapor below around 40 km. This feature is well reproduced in the reanalysis (Figures 2a–2e), also seen in high polar latitudes before *L*_S_ = 200° in Figure 4. During the time of the global dust storm (*L*_S_ = 190–230°) in MY 34, water vapor at northern polar latitudes displays a large increase in abundance above 40 km (Figures 2d and 2e), with the gradient of increase displaying a good match between the independent SPICAM profiles and the reanalysis (Figure 4d).

Approaching perihelion, the general trend is for the water vapor to be in greater abundance in the lower atmosphere, with the transition to lower values in the middle atmosphere occurring between 40 and 60 km (Figures 2g–2i). The transition is again in very good agreement between the independent SPICAM profiles and the reanalysis, at northern polar latitudes (Figure 2g) and equatorial latitudes (Figure 2h). The maximum abundance of water vapor also displays similar levels peaking between 150 and 200 ppmv for the independent SPICAM profiles and the reanalysis. The free‐running GCM tends to overpredict water vapor abundance in particular at high altitudes, a common feature in GCMs that include supersaturation. While the SPICAM profiles unfortunately do not cover the whole altitude range that is possible to simulate in the reanalysis, a double peak structure in Figure 2g is seen both in the SPICAM profile and the reanalysis with very good agreement in altitude. The validation through comparison against available independent SPICAM water vapor profiles indicates the reanalysis data set is the optimal data set to use for investigating the water cycle and captures realistic vertical structures in the atmospheric water cycle.

## 5 Results

**Figure 3:**
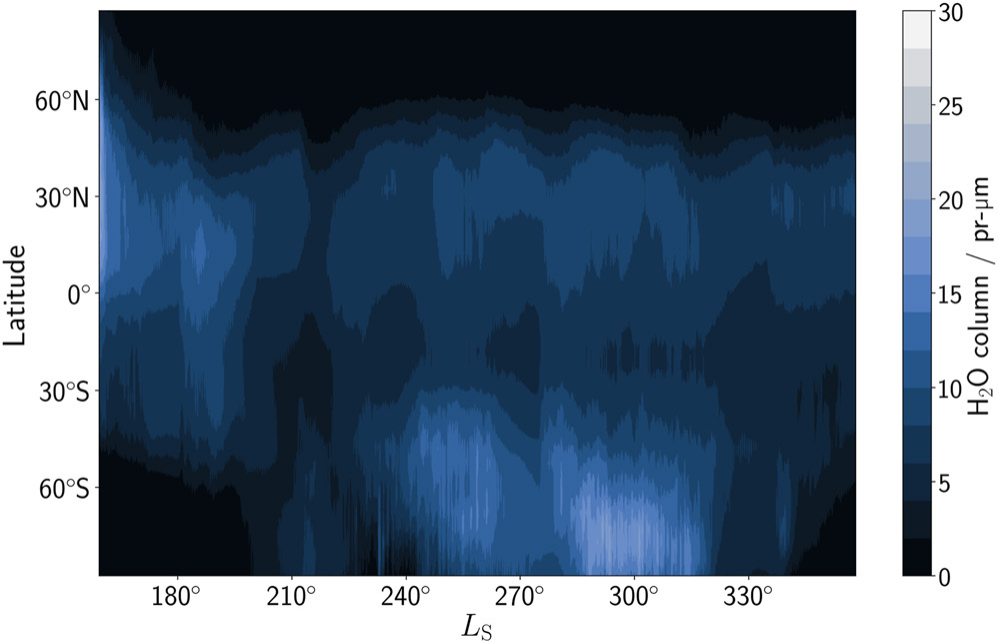
Zonally‐averaged water vapor column in the water reanalysis covering *L*_S_ = 159–358° in Mars Year 34.

Figure 3 shows the zonal average of the water vapor column in the water reanalysis. The characteristic seasonal evolution of the water vapor column during the second half of a MY is reproduced, with polar winter in the northern hemisphere containing virtually no water vapor, and the southern summer sublimation of water vapor from the southern polar cap evident in the southern polar region around perihelion. Interested readers on how the reanalysis compares to a free‐running GCM or when only temperature/dust observations or temperature/dust/water vapor column observations only are assimilated can refer to the figures in Supporting Information S1. During the global dust storm in MY 34, the uncertainty in retrieved NOMAD water vapor column is known to be much larger because of local extreme dusty conditions (Crismani et al., 2021). To take this into account, the ratio of uncertainties between the retrievals and the model is increased by a factor of 2 to appropriately account for the increased uncertainty in the water vapor column retrievals during *L*_S_ = 185–220° MY 34. The column abundance of water vapor in the reanalysis during this time period is around 25%–50% higher than the retrievals themselves, confirming the conclusion in Crismani et al. (2021) that the NOMAD nadir total column retrievals did not observe the whole atmospheric column.

**Figure 4:**
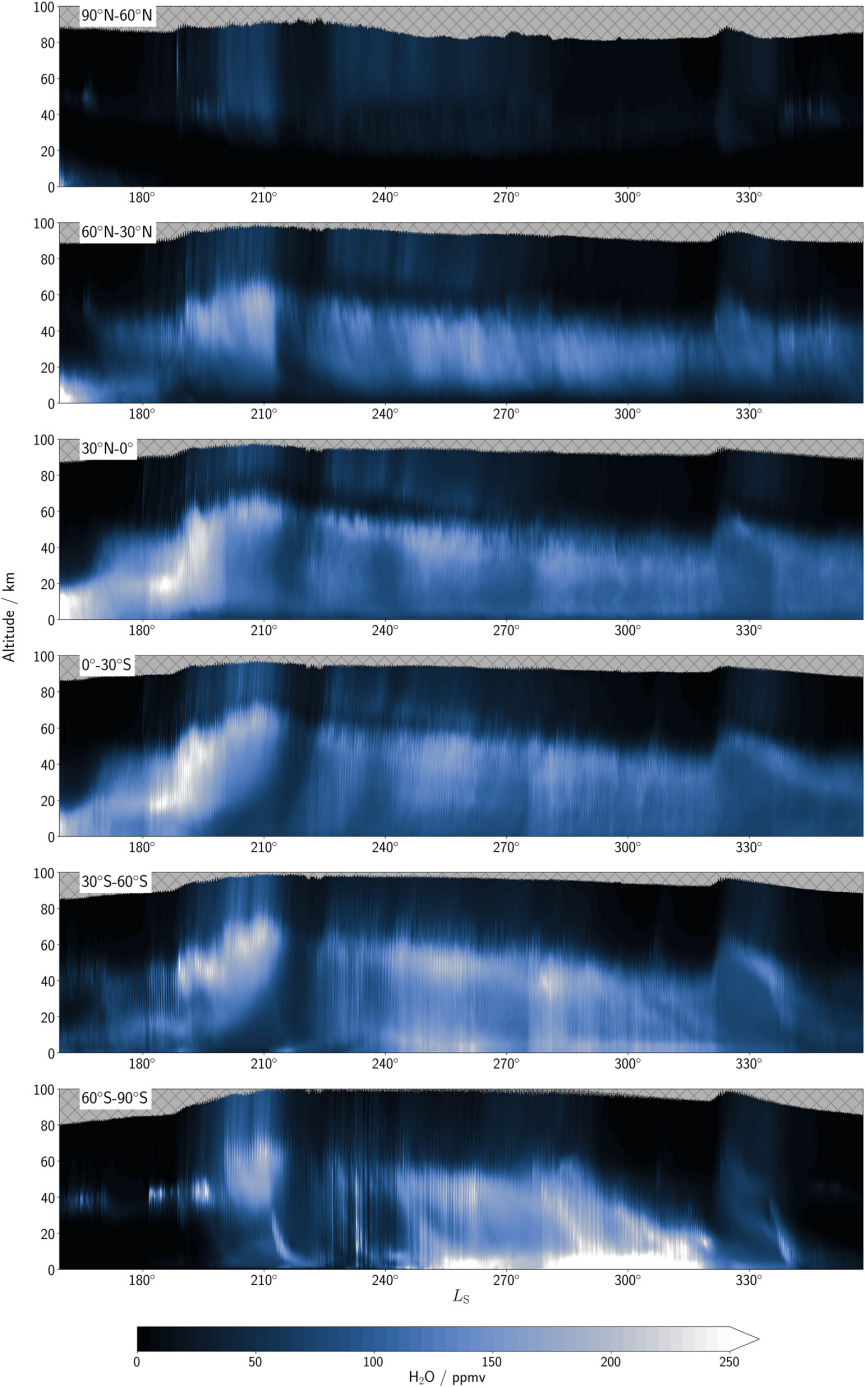
Zonally‐averaged water vapor profiles averaged over 30° latitudinal bands in the water reanalysis covering *L*_S_ = 159–358° Mars Year 34.

With the inclusion of water vapor profiles from both the NOMAD and ACS instruments, additional constraints on the vertical distribution alongside the column total are now feasible. The vertical distribution of water is highly variable and dependent on the season and latitudinal location, as illustrated in Figure 4 which displays the 30° zonally averaged vertical profile of water vapor at a selection of different latitude bands (30° latitudinal means) over time. Stand‐out features identifiable are the influence of the global dust storm (e.g., Kass et al., 2020) that occurred from *L*_S_ = 180–225° and the intense southern summer regional dust storm, also known as a C storm (Kass et al., 2016), from *L*_S_ = 320–335° which both provided an increased abundance of water vapor to the upper atmosphere at all latitudes including the northern polar region compared to years where no intense dust storms are observed, along with the perihelion water “pump” season previously identified by Shaposhnikov et al. (2019) and observed in the ExoMars TGO vertical profiles of water vapor (Aoki et al., 2019; Fedorova et al., 2020; Villanueva et al., 2021). Sublimation of water vapor from the southern polar cap in southern summer is also evident from *L*_S_ = 255–315° in the 60°S–90°S latitude band. In the 90°N–60°N latitude band, water vapor is extremely low in abundance below 25 km during the second half of MY 34, with evidence of water vapor below 25 km at the end of spring at the start of the simulation. Future assimilation studies will expand the reanalysis to cover the first half of MY 35 when further profiles are made available, where increased levels of water are expected to be found at northern polar latitudes because of the different season.

The assimilation method can be used as an effective tool for identifying potentially spurious retrievals, with a sharp short‐lived increase in water vapor centered on 80 km at around *L*_S_ = 190° in the 90°N–60°N latitude band (Figure 4) indicative of such a retrieval. On further investigation, the daily cycle of solar insolation coupled with topography gradients seem to create “ripples” in the water vapor vertical distribution that could suggest this to be a true event (further investigation is warranted but out of the scope of this paper). The following results that investigate the global water distribution in the reanalysis are applicable to MY 34 only, which featured a global dust storm and strong C storm.

### 5.1 Global Transport

**Figure 5:**
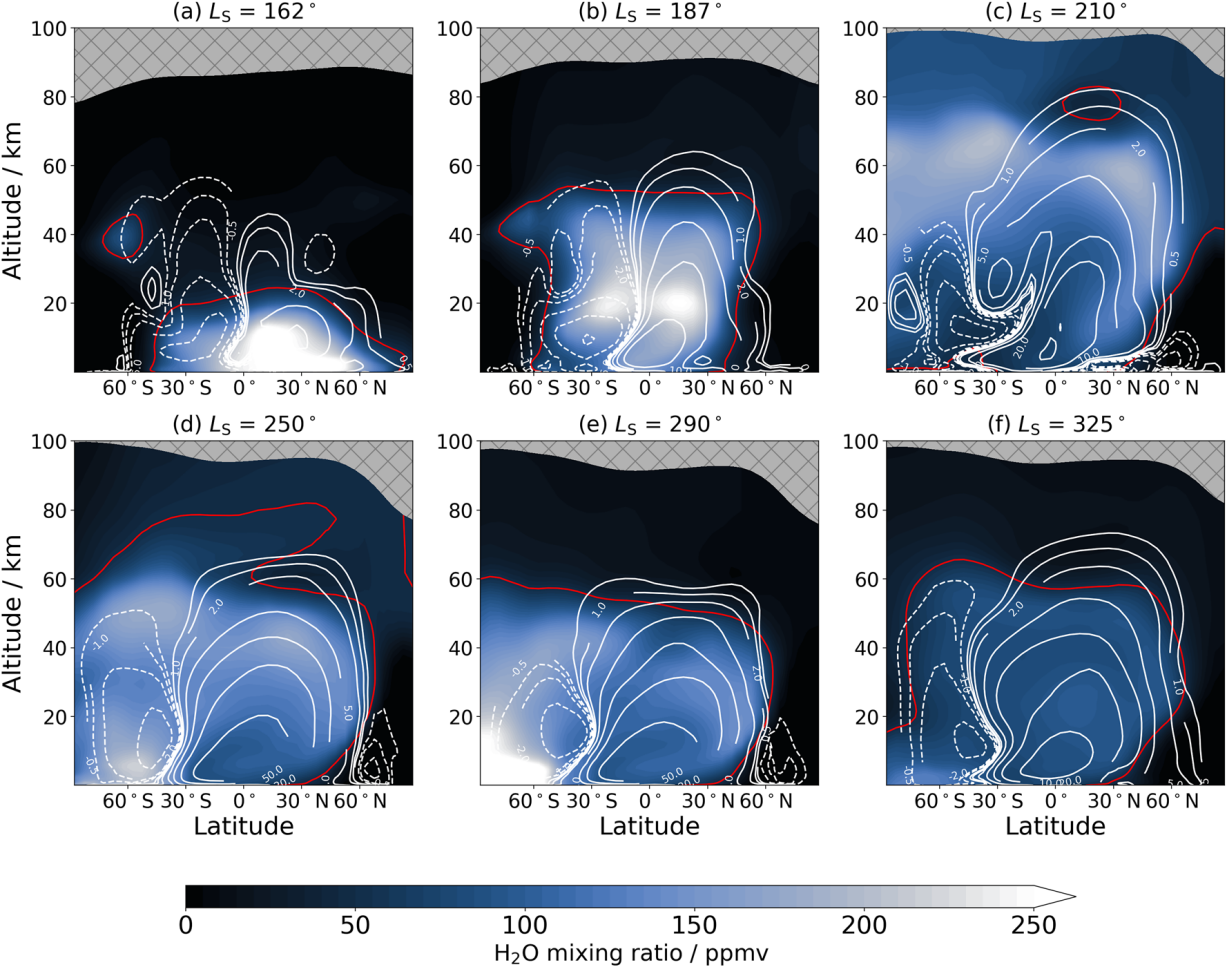
Zonally‐averaged latitude‐altitude cross‐sections of the water vapor vertical distribution at six different times of Mars Year 34 covered by the reanalysis. White contours indicate the mean meridional circulation with solid/dashed representing clockwise/anti‐clockwise motion. The mean meridional circulation contours have pseudo‐logarithmic values of (0.5, 1, 2,5, 10, 20, and 50) × 10^8^ kg s^−1^. The red line is an approximate indicator for the hygropause (50 ppmv). The data are averaged over a 10‐sol window centered on the time stated.

**Figure 6:**
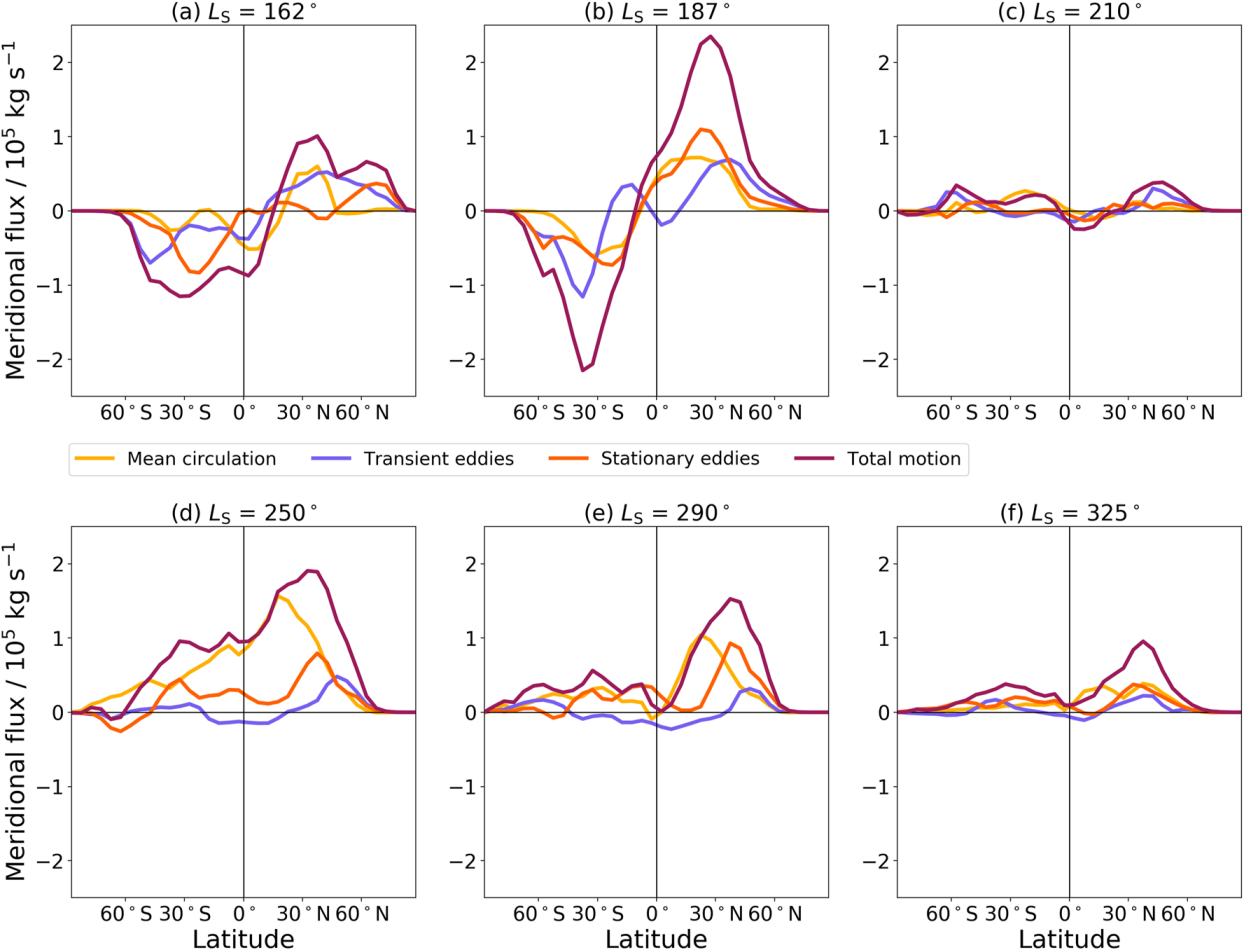
Column‐integrated meridional flux of water vapor for four different transport modes at six different times of Mars Year 34 covered by the reanalysis. The data are averaged over a 20‐sol window centered on the time stated.

With constraints imposed directly on the atmospheric temperature state and water vapor vertical distribution and total column, the global transport of water vapor can be investigated. Figure 5 displays the zonally‐averaged vertical distribution of water vapor at six different periods in MY 34, while Figure 6 shows the column‐integrated meridional flux of water vapor that has been decomposed into four different transport modes in a similar manner to Steele, Lewis, Patel, Montmessin, et al. (2014). Direct comparison of the results found here to those found by Steele, Lewis, Patel, Montmessin, et al. (2014) are complicated by several factors: the results here use a microphysical scheme and constrain the vertical profile of water vapor using available retrievals, both of which are likely to produce a different vertical distribution of water vapor. Also, the time period studied in this investigation includes a global dust storm. The dynamics in the reanalysis are also better constrained since in this study we assimilate MCS and ACS temperature profiles which extend up to around 80 km, whereas Steele, Lewis, Patel, Montmessin, et al. (2014) assimilated TES temperature profiles which only extended from the near‐surface to a peak altitude of around 40 km. With the inclusion of ACS temperature profiles alongside MCS temperature profiles, the local time at which the temperature profiles have the most impact on the temperature field are expanded compared to the relatively fixed local times of TES retrievals.

#### 5.1.1 Global Dust Storm, Growth Phase (*L*_S_ = 159–200°)

At the start of the simulation, water vapor is generally confined to below 20 km at all latitudes northward of 30°S as seen in Figures 4 and 5a. From 60°S to 90°S, water vapor at the start of the simulation is only seen in a band at around 40 km altitude. This water vapor layer is linked to the anti‐clockwise cell transporting water vapor from lower latitudes into this region from the upwelling branch at around 10°S (Figure 5a) and a small clockwise cell at lower latitudes at around 50°S restricting further downwelling of the water vapor layer. Figure 6a indicates that most of the southward transport is split between transient and stationary eddies rather than the mean meridional circulation. During the onset of the MY 34 global dust storm, wave activity controls the transport of water poleward of 30°N and 30°S (Figure 6b), with wave activity (transient and stationary eddies) generally acting to transport water vapor polewards in each hemisphere in agreement with what was found by Steele, Lewis, Patel, Montmessin, et al. (2014). In equatorial latitudes the mean meridional circulation is generally transporting water northwards with a switch southwards at around 15°S (Figure 6b). The hygropause (defined here as the highest point at which water is present at 50 ppmv as this generally coincides with the location of the rapid decrease in water vapor content) has risen considerably since the start of the simulation and is beyond 50 km during the initiation phase of the global dust storm (Figure 5b). The circulation cells have expanded as a result of the increased atmospheric temperature from the increased dust loading, and as a consequence the meridional flux has also increased in magnitude reaching peak values above 2 × 10^5^ kg s^−1^ (Figure 6b).

In Figure 5b, peak water vapor volume mixing ratio is now at an altitude of around 20 km for the main bulk of water vapor compared to near the surface before the initiation phase of the global dust storm (Figure 5a). The mean meridional circulation is most active at around 30°N and 30°S in transporting water vapor northwards and southwards respectively, with wave activity still transporting more water vapor poleward than the mean meridional circulation in both hemispheres by a factor of two (Figure 6b). Water vapor is also transported to polar regions, but largely confined to altitudes above 40 km, with a slight asymmetry toward increased water abundance over the southern polar region compared to the north (Figure 5b).

#### 5.1.2 Global Dust Storm, Mature Phase (*L*_S_ = 200–245°)

During the mature phase of the global dust storm, the hygropause is now located above the top of the reanalysis, that is, above 95 km (Figure 5c). The expansion of the hygropause level during the global dust storm is in agreement with the abundance of water retrieved by the NOMAD (Aoki et al., 2019) and ACS (Fedorova et al., 2020) data sets. As the subsolar point begins to move southward, the clockwise circulation cell has expanded further (Figure 5c) and is beginning to dominate the anti‐clockwise cell. A large absolute reduction in meridional flux is evident in Figure 6c, but this is not linked to any reduction in the strength of the different transport processes (wave activity and circulation) but because of reduced atmospheric water vapor content (Figure 3). In low southern latitudes, northward transport by stationary eddies linked to increased transport over Argyre planitia is in good agreement with results found in Steele, Lewis, Patel, Montmessin, et al. (2014), Figure 13. The increased dust loading when compared to the global transport studied in Steele, Lewis, Patel, Montmessin, et al. (2014) is likely to contribute to different patterns in transient eddies and mean meridional circulation displayed in Figure 6c. The latitudinal pattern of stationary eddies is however in good agreement between both MYs studied, indicating that the global dust storm had a larger influence in altering the transient and mean meridional circulation transport modes.

Water vapor at an abundance above 50 ppmv is now found at all latitudes above 40 km including the northern polar latitudes (Figure 4), with only the cold northern polar winter region containing less water vapor. After *L*_S_ = 215° in the 60°S–90°S latitude band, a layer of water vapor begins to move progressively lower in altitude as time progresses (Figure 4). This transport is initially blocked by a small clockwise cell located at around 20 km poleward of 60°S (Figure 5c) which, during the decay phase of the global dust storm, diminishes as the downwelling branch of the anti‐clockwise cell begins to dominate the vertical transport of water vapor.

#### 5.1.3 Southern Summer (*L*_S_ = 245–295°)

As perihelion approaches, the anti‐clockwise cell continues to strengthen (Figure 5d), which results in the hygropause altitude returning to lower altitudes of 60–80 km southward of 60°S. The clockwise cell still maintains a high hygropause altitude for the majority of latitudes northward of 60°S. The global transport of water vapor is largely switching to northward movement (Figure 6d), as the subsolar point moves further southward and the upwelling branch subsequently shifts further south toward 40°S (Figure 5d). The strongest meridional flux is at high northern latitudes, reaching peak values over 2 × 10^5^ kg s^−1^, similar to the initiation phase of the global dust storm in Figure 6b, and begins to be dominated by stationary waves and mean meridional circulation later in the time period (Figure 6e).

The hygropause level gradually descends at most latitudes (Figure 4) and by *L*_S_ = 290° the hygropause is below 60 km at all latitudes (Figure 5e). The anti‐clockwise cell is close to the south pole and restricts northward transport of water vapor away from the pole during peak sublimation of water vapor from the southern polar cap, in agreement with results found by Steele, Lewis, Patel, Montmessin, et al. (2014). During this time most of the water vapor is located from 30°S to 90°S (Figure 4). The peak in northward flux of water vapor is strongly linked to enhanced transport by stationary eddies at around 30°N from the start of this time period (Figure 6d) to the end (Figure 6e).

#### 5.1.4 Southern Autumn (*L*_S_ = 295–358°)

During this time period, the rapid drop‐off in water vapor abundance with altitude is located at around 40 km at most latitudes by *L*_S_ = 320° (Figure 4) before water vapor is found in increased abundance a week later at a much higher altitude. This increase was linked to the particularly intense southern summer regional dust storm in MY 34 (Chaffin et al., 2021), which again strengthened the circulation cells (Figure 5f) and lifted the rapid drop‐off in water vapor above 60 km once more predominantly at southern polar latitudes.

Transport of water vapor is still predominantly northward (Figure 6f) but weaker in strength than earlier in the season (Figure 6e), with transport of water vapor in the southern polar region dominated by stationary eddies (Figure 6f). In the northern polar region, stationary eddies and the mean meridional circulation have an equal impact on northward transport of water vapor. The northern polar anti‐clockwise cell present before the C storm (Figure 5e) is diminished during and after the C storm as the subsolar point once again moves northwards and by the end of the year is reverting to the split cell structure seen at the start of the simulations. After around *L*_S_ = 315°, sublimation from the southern polar cap stops and, by the end of the year, water vapor is largely confined to mid‐latitude locations (Figure 4) in agreement with Steele, Lewis, Patel, Montmessin, et al. (2014).

### 5.2 Supersaturation

The existence of supersaturation in the martian atmosphere, observed by Maltagliati, Montmessin, et al. (2011) in select SPICAM vertical profiles of water vapor, has implications for the long term inventory of water on Mars and how much has escaped in its past. The water vapor observed by Maltagliati, Montmessin, et al. (2011) above the hygropause raises questions on possible variability in hydrogen escape that is facilitated after photodissociation. Water vapor in the upper mesosphere on Mars (Belyaev et al., 2021) and supersaturation have also been found in more recent profiles retrieved by the ACS instrument (Fedorova et al., 2020). The reanalysis allows us to systematically map and investigate supersaturation globally and at different local times, which is not possible with retrievals alone as a result of spatiotemporal restrictions. Navarro et al. (2014) investigated briefly the supersaturation state of the atmosphere in the LMD Mars GCM and found reasonable agreement with the SPICAM retrievals displayed by Maltagliati, Montmessin, et al. (2011). The reanalysis here uses the same microphysical package but now also has the addition of temperature and water profile assimilation to constrain further both of these aspects of the martian atmosphere. One limitation of the current reanalysis data set is that only total dust column was assimilated, so the degree of supersaturation is likely to be an upper limit especially high in the atmosphere. Nevertheless, ACS coincident water vapor and temperature profiles have confirmed supersaturation does exist high in the martian atmosphere throughout the dusty season (Fedorova et al., 2020).

**Figure 7:**
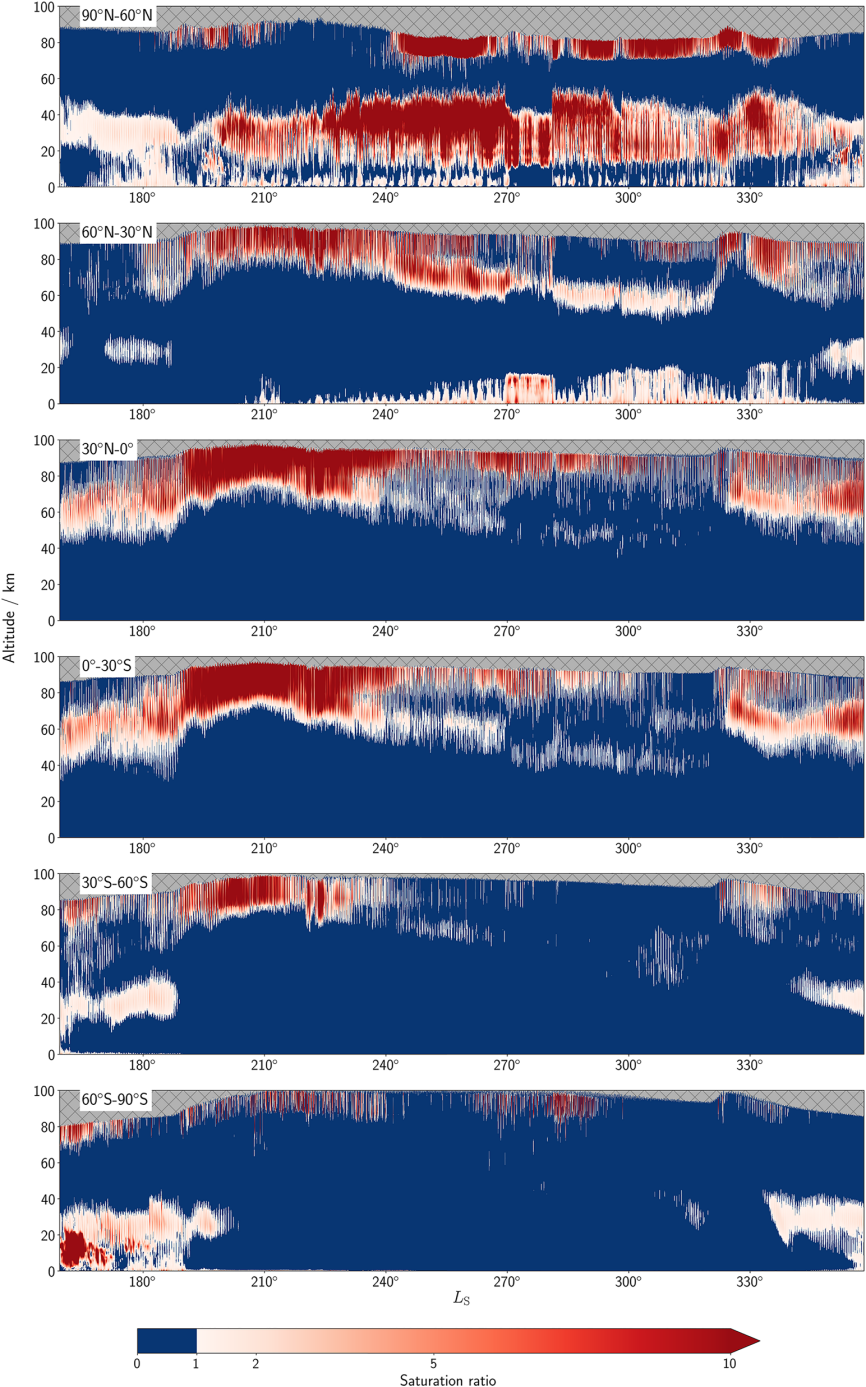
Hourly profiles of the zonally‐averaged saturation ratio averaged over 30° latitudinal bands in the water reanalysis covering *L*_S_ = 159–358° Mars Year 34.

Figure 7 displays the 30° latitudinal‐average vertical saturation ratio profile at 0° longitude every hour, calculated via the Goff‐Gratch equation also used by Fedorova et al. (2020). Large portions of the lower atmosphere (below around 40 km) are subsaturated no matter what time period or local time is investigated, in particular for latitudes covered by 30°N–30°S. Several features identified by the ACS retrievals (Fedorova et al., 2020) are reproduced by the reanalysis. These include the split supersaturation near‐surface structure at northern polar latitudes around *L*_S_ = 180° with a supersaturated atmospheric portion at around 20–40 km (and return to a subsaturated atmosphere above around 40 km). This supersaturated layer is also found at a similar time of year (*L*_S_ = 180°) in the southern polar region. In the southern hemisphere, the ACS retrievals identified supersaturation present at lower altitudes toward the equator, with a supersaturation boundary around 80 km in the southern polar region and around 60 km closer to the equator around *L*_S_ = 200–225°, with the water reanalysis showing a matching trend. We show in the reanalysis that this feature is apparent for both hemispheres. Also at this time, at around 75°S, there is a peak in supersaturation at around 75 km in the ACS retrievals shown in Fedorova et al. (2020) Figure 2d. The water reanalysis shows small pockets of supersaturation centered on *L*_S_ = 210° in the 60°S–90°S latitude band. This is similar to a feature found by Maltagliati et al. (2013) reported to be the first identification of supersaturation within a cloud, albeit at an altitude around 5 km higher than in the reanalysis and ACS retrievals. The retrievals do however come from different MYs, with the MY 34 retrievals and reanalysis here occurring during a global dust storm which could potentially push the supersaturation boundary to higher altitudes as a result of the increased atmospheric temperature and vertical expansion of the circulation cells.

During the perihelion season, supersaturation at polar southern latitudes is at a much higher altitude than its northern counterpart. Southern polar latitudes during northern winter solstice were largely unobserved by TGO, and when retrievals are available again (around *L*_S_ = 275°) the spacecraft geometry means they only go as far north as around 45°N in latitude and indicate a layer of supersaturation around 45–70 km which is in reasonable agreement with the reanalysis in Figure 7. A new feature identified in the water reanalysis is a supersaturation layer centered roughly at 40 km at latitudes poleward of 60°N that corresponds to the pocket of cold air below the polar warming at higher altitudes. This layer generally persists throughout the whole of the MY 34 dust season as shown in Figure 7. During peak northern winter, this layer can extend at times down below 20 km and the structure of long periods of subsaturation followed by extended periods of supersaturation is linked to baroclinic wave activity on the edge of the northern polar hood (Lewis et al., 2016). The presence of supersaturated water vapor here has implications for the surface‐atmosphere exchange of water vapor between northern polar winter and mid‐latitude locations and is explored further in the next section. A feature reported by Fedorova et al. (2020) is a discrete layer of supersaturation at southern polar latitudes (20–40 km altitude) toward the end of MY 34 at around *L*_S_ = 315°. This discrete layer is also present in the water reanalysis (Figure 7), with additional insight from the extended local time coverage provided by the reanalysis indicating that this discrete layer disappears during the daytime.

**Figure 8:**
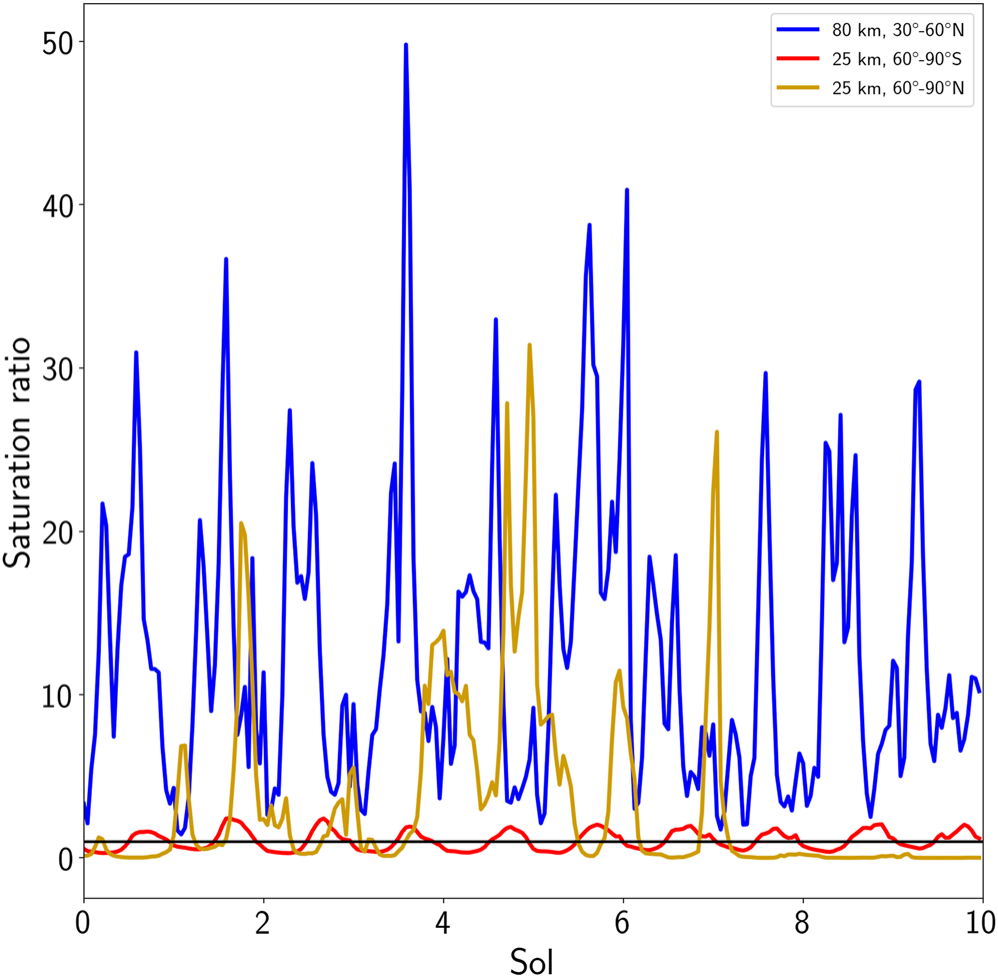
Hourly output of the saturation ratio of water vapor at the specified altitude and latitude band over a 10 sol time window centered at *L*_S_ = 210° (blue), *L*_S_ = 250° (yellow) and *L*_S_ = 345° (red) in the Mars Year 34 reanalysis. The black solid line indicates a saturation ratio of one.

Supersaturation persists throughout the diurnal cycle above 70 km across the majority of latitudes during the peak (around *L*_S_ = 204°) and well beyond the decay phase of the global dust storm in Figure 7, which will increase the amount of water that can escape the atmosphere during this time period. Examples of the diurnal variations in supersaturation are shown in Figure 8, with the primary modes of supersaturation in the atmosphere either being completely supersaturated throughout repeated diurnal cycles or a clear switch between supersaturation and subsaturation in the day/night cycle. A third mode that is less frequent than the two identified above is short extended periods of supersaturation covering multiple sols followed by periods of subsaturation covering several sols. This is prominent at northern polar latitudes, from *L*_S_ = 230–270°, in particular during northern polar winter, from around 20–40 km in the atmosphere (see Figure 7) and are influenced by the transient eddies or localized weather events that control the water vapor distribution in this spatiotemporal region.

The saturation ratio can reach high values at high altitudes, although this refers to very small values of water vapor abundance that exceed a threshold value of saturation which is also extremely small. The reanalysis does not currently assimilate dust profiles and tends to underpredict the height at which dust can reach in the atmosphere. Dust simulated at higher altitudes more in line with observations would likely reduce the saturation ratio as the water vapor present would be able to phase change into ice. At lower altitudes where a diurnal structure is seen the results are unaffected by the previous point. The impact of the global dust storm and the C storm later in the year (*L*_S_ = 326.1–333.5°) was to force the boundary between subsaturated and supersaturated water vapor higher in the atmosphere from 60°N to 60°S (Figure 7), with enhanced water loss from the atmosphere also having been observed (Chaffin et al., 2021) and modeled (Holmes et al., 2021) during this time. The supersaturated layer at latitudes poleward of 60°N was largely unaffected by the global dust storm, but the supersaturated layer is shifted to a slightly higher altitude by the C storm in Figure 7. While the global dust storm seemed to have negligible impact on the saturation state of the atmosphere from 60°S to 90°S, the C storm resulted in the disappearance of the supersaturated layer that was present from 20 to 40 km before this major dust event as a result of the additional heating of the atmosphere through the increased presence of dust.

Whether the mapping of supersaturation discussed here is typical of any MY requires a similar assimilation for a non‐global dust storm year, which will be possible in future by assimilating NOMAD water vapor profiles from MY 35 (when they become available) alongside available ACS water vapor and temperature profiles.

### 5.3 Northern Polar Water Vapor During the Northern Winter Season

**Figure 9:**
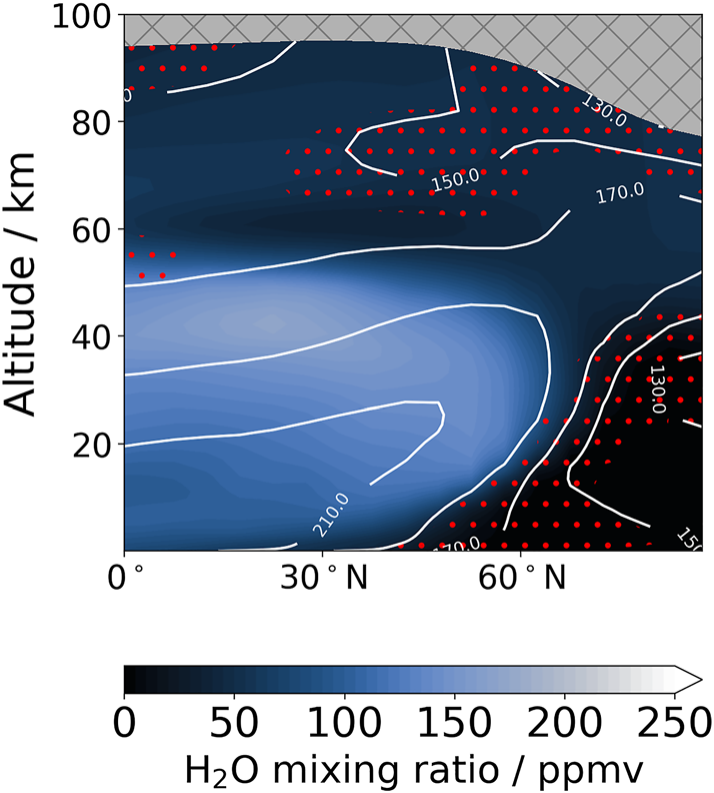
Zonally‐averaged latitude‐altitude cross‐section of the water vapor vertical distribution and atmospheric temperature (white contours) at *L*_S_ = 250° Mars Year 34 in the reanalysis. Red dots indicate presence of supersaturated water vapor. The data are averaged over a 10‐sol window centered on the time stated.

From past total column observations of water vapor (Smith, 2004; Trokhimovskiy et al., 2015), the northern polar region during northern polar winter is expected to contain extremely low levels of water vapor (less than 1 pr‐μm). The results in Figure 3 are in agreement with the total column retrievals available, but while the total column abundance is extremely low, the vertical profile of water vapor poleward of 60°N varies from around 0–50 ppmv (Figure 5d). The abundance of water vapor in the reanalysis is also in agreement with independent retrievals from SPICAM (Fedorova et al., 2021). Figure 9 displays the vertical structure of northern hemisphere water vapor and atmospheric temperature during northern polar winter focused on a 10 sol period around *L*_S_ = 250°. The increased water vapor abundance (above 50 ppmv) directly over the north pole above around 40 km is linked to the polar warming and meridional circulation pattern during this time of year in which water vapor is transported northward by the cross‐hemisphere clockwise cell (Figure 5d). The cold polar winter vortex has long been thought to be a barrier to chemical transport (Waugh et al., 2016), with no previous evidence of transport of water vapor into northern polar latitudes during northern polar winter even when assimilating water vapor column data as shown in Steele, Lewis, Patel, Montmessin, et al. (2014), Figure 12. The water reanalysis in this study, which includes supersaturation of water vapor, shows evidence of water breaking through into the polar vortex region, with water vapor from 20 to 50 km during this spatiotemporal location regularly in a supersaturated state as shown in the top subplot of Figures 7 and 9. This finding has implications for surface‐atmosphere exchange of water vapor during northern polar winter that could therefore be larger than previously thought with increased transport into the northern winter polar vortex and toward the northern polar cap.

**Figure 10:**
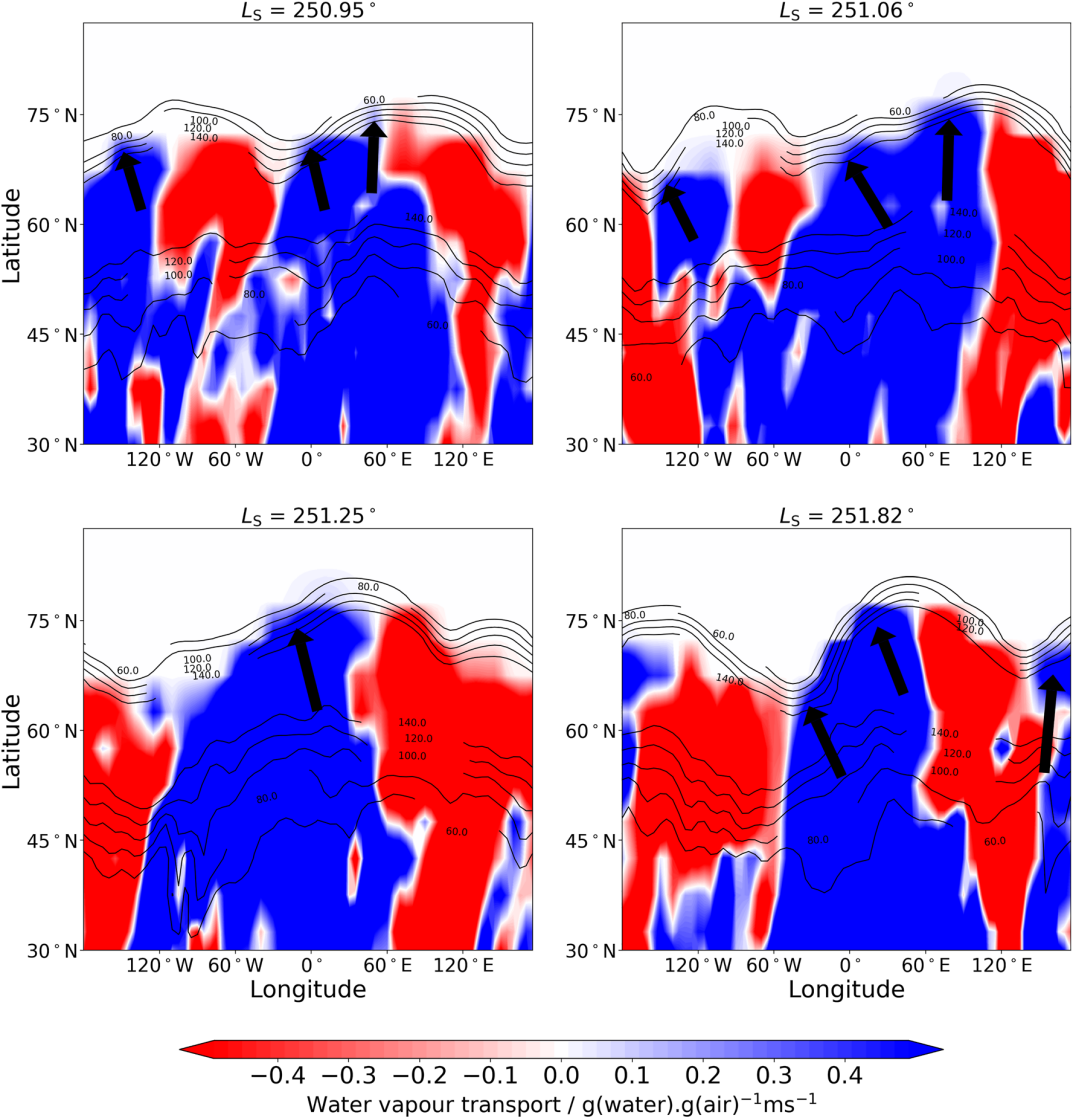
Longitude‐latitude maps at 35 km of the meridional transport of water vapor in the water reanalysis at the time specified above each subplot. Blue/red indicates transport to the north/south. Black contours indicate the zonal wind at 35 km. Black arrows indicate where water vapor is crossing the northern winter polar vortex boundary.

The analysis of different transport modes in Figure 6d suggests that poleward of 60°N the water is transported favorably by transient eddies and stationary waves rather than the mean meridional circulation. A previous reanalysis of TES temperature profiles and water vapor column retrievals (Steele, Lewis, Patel, Montmessin, et al., 2014) did not yet include a microphysics package and therefore was not able to simulate supersaturated water vapor and reveal this feature. Alternatively, the lack of a global dust storm in the previous reanalysis could also have been a factor. Figure 10 shows four snapshots of the meridional flux of water vapor at 35 km in the northern hemisphere around *L*_S_ = 250°. The boundary of the northern polar vortex can be interpreted as the large gradient in zonal wind, which for Figure 10 will roughly correspond to the 100 ms^−1^ contour poleward of 60°N. A zonal wavenumber 2 stationary wave is clearly evident in the pattern of meridional transport, with northward transport of water vapor across the northern polar winter polar vortex boundary occurring at different longitudes depending on the local time. This northward transport appears to occur throughout the majority of the northern polar winter season as can be seen in the top subplot of Figure 7, indicating that the northern winter polar vortex is not as strong a boundary for tracer transport as previously thought. If this feature occurs every MY during northern polar winter, it has potential implications for the long term inventory of the northern polar cap.

Whether similar breaches of water into the southern polar winter vortex are apparent during southern winter requires a water reanalysis of this particular season, which will be possible in future when NOMAD water vapor profiles from MY 35 become available to assimilate along with available ACS water vapor profiles.

## 6 Conclusions

The transport of water vapor throughout the martian atmosphere is investigated in a water reanalysis that assimilates water vapor profiles from multiple spacecraft instruments to constrain the vertical distribution as well as the column total. Assimilated temperature profiles also contain a wider range of local times in the MY 34 water reanalysis than in previous assimilation studies, providing additional constraints on the flux of water vapor throughout time. The altitude of the hygropause is controlled by the occurrence of the major dust events in MY 34 with the influence extending to both polar regions. New evidence of water vapor, in a supersaturated state, breaking into the northern winter polar vortex is found in the MY 34 water reanalysis. This finding has implications for the surface‐atmosphere exchange of water between the northern polar cap and the large scale atmosphere (via transport of water vapor). The results here suggest that the seasonal flux of water vapor into the northern polar cap could be larger than previously though. This finding would also have a potential impact on how much water was present in the past atmosphere of Mars if the breaking of supersaturated water into the northern polar vortex is happening repeatedly each MY.

Results on the flux of water vapor throughout the dusty season are largely consistent with previous studies, although differences exist primarily as a result of the additional constraints on the vertical distribution of water vapor in the MY 34 water reanalysis, the simulation of supersaturation of water vapor, and a global dust storm and intense southern summer regional dust storm occurring in the MY 34 water reanalysis. Up to this point in time, supersaturation in the martian atmosphere has only been seen in select atmospheric profiles that suffer from limitations in space and time. Mapping of supersaturation is expanded globally using the MY 34 water reanalysis data set. Supersaturated water vapor is found above around 60 km for most of the dusty season in MY 34, with distinct layers lower in the atmosphere. The global dust storm and southern summer regional dust storm, in particular, forced water vapor to higher altitudes and into a supersaturated state based on the local temperature. At these altitudes it is more likely to escape from the atmosphere, and the reanalysis indicates that this happened across all latitudes. Supersaturated layers at lower altitudes generally show a diurnal structure as a result of the daily temperature cycle, whereas the high altitude layers are supersaturated for long periods of time. Understanding of the distribution of supersaturated water vapor and how it differs in a MY without a global dust storm is important to provide additional constraints on the amount of water lost from the martian atmosphere over time, and will be possible in future when water profiles from MY 35 become available.

A general problem for scientific study of Mars utilizing a Mars GCM is the lack of wind data. The MY 34 water reanalysis in this study takes advantage of the assimilation method to constrain winds as much as possible by assimilating several sources of thermal data from multiple spacecraft instruments. Results on the transport of water throughout the atmosphere in the MY 34 water reanalysis have an advantage, therefore, over pure GCM modeling studies that rely solely on simulated winds. Validation of lower atmosphere winds would provide increased confidence in the evolution of the water cycle represented by the water reanalysis, and, therefore, allow us to provide better estimates of how much water has been lost from the martian atmosphere and the potential for habitability in its past. The reanalysis created by this study is a publicly available data set that can be used for multiple atmospheric studies covering this time period, containing physically consistent variables that can be used to investigate the evolving 4‐D martian atmosphere.

## Supporting information

Supporting Information S1

Figure S1

Figure S2

## Data Availability

The simulation data used in this study are publicly available via the Open Research Data Online (ORDO) data repository as part of the OpenMARS database (Holmes et al., 2022).
