# Supplementary material for: Global Variations in Water Vapor and Saturation State Throughout the Mars Year 34 Dusty Season
Source: J Geophys Res Planets. 2022 Oct 21;127(10):e2022JE007203. doi: 10.1029/2022JE007203 (PMC9788072; doi:10.1029/2022JE007203)
Supplement: Supplementary file 1 — Supporting Information S1 [file JGRE-127-e2022JE007203-s003.pdf]

JOURNAL OF GEOPHYSICAL RESEARCH

Supporting Information for "Global variations in water vapour and saturation state throughout the Mars Year 34 dusty season"

J. A. Holmes[1], S. R. Lewis[1], M. R. Patel[1,2], J. Alday[1,3], S. Aoki[4,5], G. Liuzzi[6,7], G. L. Villanueva[6], M. M. J. Crismani[8], A. A. Fedorova[9], K. S. Olsen[3], D. M. Kass[10], A. C. Vandaele[5], and O. Korablev[9]

[1]School of Physical Sciences, The Open University, Milton Keynes, MK7 6AA, UK

[2]Space Science and Technology Department, Science and Technology Facilities Council, Rutherford Appleton Laboratory, Harwell Campus, Didcot, Oxfordshire, OX11 0QX, UK

[3]Department of Physics, University of Oxford, Oxford, UK

[4]Institute of Space and Astronautical Science, Japan Aerospace Exploration Agency, Kanagawa, Japan

[5]Royal Belgian Institute for Space Aeronomy, Belgium

[6]NASA Goddard Space Flight Center, Greenbelt, MD, USA

[7]Department of Physics,American University, Washington, DC, USA

[8]California State University, San Bernardino, Department of Physics, CA USA

[9]Space Research Institute of the Russian Academy of Sciences (IKI RAS), Russia

[10]Jet Propulsion Laboratory, California Institute of Technology, USA

Contents of this file

1. Text S1

---

2. Figures S1 and S2

Introduction The supporting information displays a comparison of the reanalysis investigated in the mansucript with a free-running GCM, a reanalysis of temperature and dust observations only, and a reanalysis of temperature, dust and water vapour column observations only. These comparisons help to identify the impact of the different assimilation products on the water reanalysis.

Text S1. A zonally-averaged comparison of the water vapour column in the water reanalysis with the additional simulations is shown in Figure S1. Assimilation tends to dry out the water cycle simulated in the free-running GCM, while a shift in the southern summer peak sublimation of water vapour is seen when only temperature and dust observations are assimilated.

A comparison of the vertical distribution of water in the water reanalysis with the additional simulation of temperature/dust and water column only is displayed in Figure S2. The figure demonstrates that while the addition of water vapour profiles does not have a large impact on the water vapour column (Figure S1) they have an obvious impact on the vertical structure of water vapour, in particular around perihelion (Figure S2d). Note that Figure S2 displays zonal averages and also short-term time averages, so the difference between the water reanalysis and the temperature/dust and water column only reanalysis may be larger at the precise time and location of the water vapour profiles, with their effect potentially smoothed out in time and through the zonal averaging.

Figure S1. Zonally-averaged water vapour column difference in the free-running GCM (top), temperature/dust only reanalysis (middle) and temperature/dust/water column only reanalysis (bottom) when compared to the reanalysis ($L_S = 159\text{-}358°$ in MY 34). Black contours indicate the zonally-averaged water vapour column for each separate simulation. Blue/red shading indicates the reanalysis is drier/wetter than the additional simulations.

Figure S2. Zonally-averaged latitude-altitude cross-sections of the difference in water vapour vertical distribution at six different times of MY 34 when comparing the water reanalysis to the temperature/dust/water column only reanalysis. Black contours indicate the water vapour volume mixing ratio distribution in the temperature/dust/water column only reanalysis. The data are averaged over a 10-sol window centred on the time stated.
