# Supplementary figures and images for: Global Variations in Water Vapor and Saturation State Throughout the Mars Year 34 Dusty Season
Source: J Geophys Res Planets. 2022 Oct 21;127(10):e2022JE007203. doi: 10.1029/2022JE007203 (PMC9788072; doi:10.1029/2022JE007203)

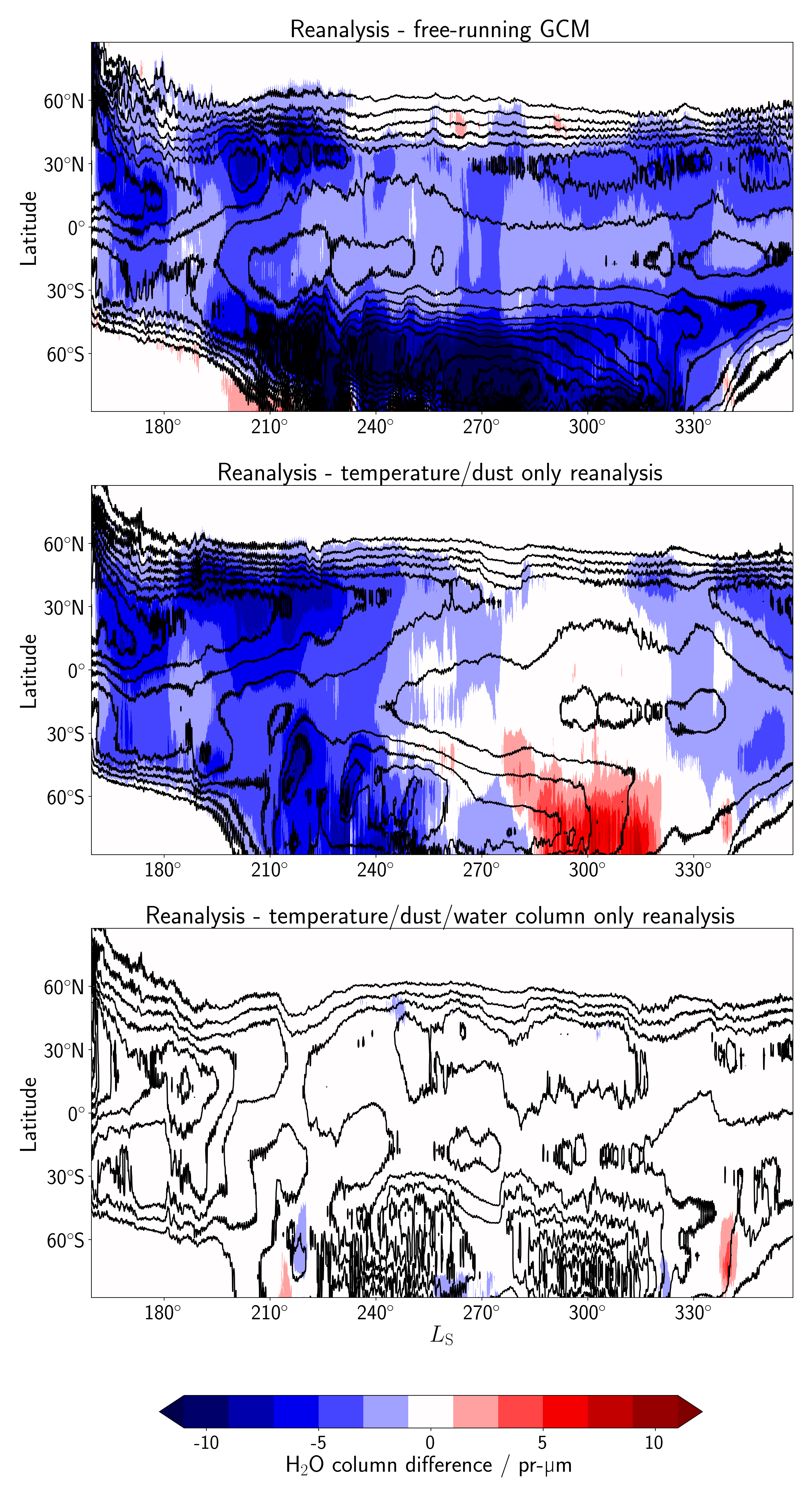

Supplement: Supplementary file 2 — Figure S1 [file JGRE-127-e2022JE007203-s001.png]

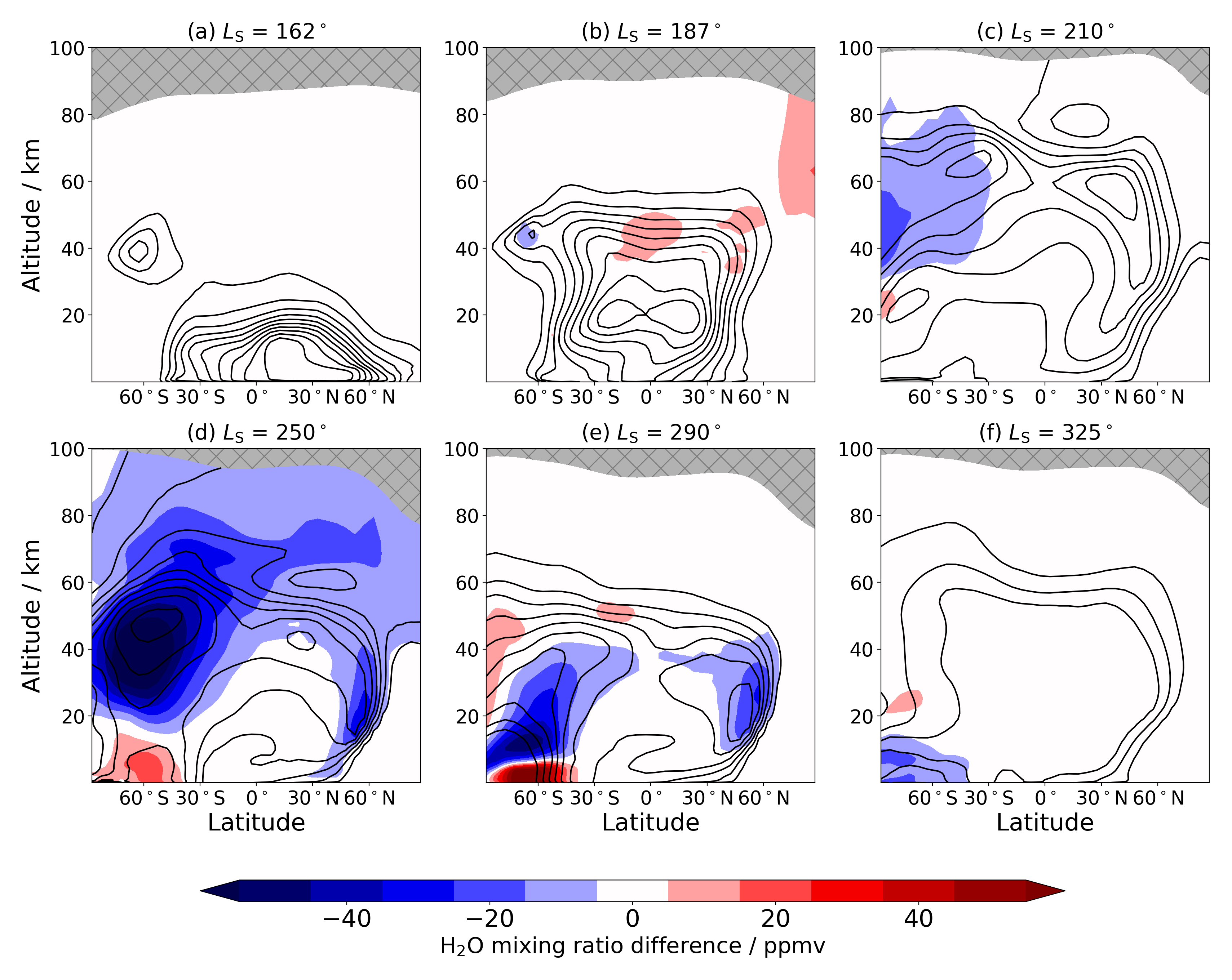

Supplement: Supplementary file 3 — Figure S2 [file JGRE-127-e2022JE007203-s002.png]
